# Formulation Engineering of Oral Semaglutide Tablets: Unleashing Gastric Intestinal Permeation with Sodium Caprate

**DOI:** 10.3390/pharmaceutics18060680

**Published:** 2026-05-29

**Authors:** Do-Hyub Kim, Sung-Kwan Hwang, Ji-Hyeon Yoon, Dong Hee Na, Young-Joon Park, Yoon-Jee Chae, Ji-Eun Chang, Joo-Eun Kim

**Affiliations:** 1 Department of Biopharmaceutical Chemistry, Kookmin University, Seoul 02707, Republic of Korea; hyufree@naver.com (D.-H.K.); o_okk0217@naver.com (J.-H.Y.); 2 MFC Co., Ltd., 35 Cheongwonsandan 7-gil, Mado-myeon, Hwaseong-si 18543, Republic of Korea; ceo@mfcglobal.co.kr; 3 College of Pharmacy, Chung-Ang University, Seoul 06974, Republic of Korea; dhna@cau.ac.kr; 4 College of Pharmacy, Ajou University, Suwon-si 16499, Republic of Korea; parkyj64@gmail.com; 5 Department of Pharmacology, College of Medicine, Konyang University, Daejeon 35365, Republic of Korea; yjchae@konyang.ac.kr; 6 College of Pharmacy, Dongduk Women’s University, Seoul 02748, Republic of Korea; 7 Department of Biopharmaceutical Chemistry, School of Applied Chemistry, Kookmin University, Seoul 02707, Republic of Korea; 8 Department of Pharmaceutical Engineering, Kookmin University, Seoul 02707, Republic of Korea

**Keywords:** GLP-1, semaglutide, oral formulation, sodium caprate, C10 enhancer, oral peptide delivery, pharmacokinetics

## Abstract

**Background/Objectives:** Oral delivery of semaglutide (Rybelsus) relies on sodium *N*-(8-[2-hydroxybenzoyl]amino)caprylate (SNAC) to enhance peptide absorption. However, formulation constraints and SNAC’s localized gastric mechanism have prompted the exploration of alternative enhancers. This study evaluated whether sodium caprate (C10), a well-characterized medium-chain fatty acid (MCFA), could achieve systemic exposure comparable to SNAC-based formulations when co-formulated in an immediate-release (IR) tablet. **Methods:** Preformulation studies assessed the physicochemical properties and buffering capacity of C10. Mechanistic feasibility was evaluated through Caco-2 transport studies and rat pharmacokinetic (PK) trials using aqueous suspensions, comparing the concentration-dependent effects of C10 and SNAC. Based on these findings, three IR tablet architectures (monolayer, bilayer, and dry compression-coated) were developed. The optimized formulation was evaluated in beagle dogs (14 mg semaglutide) and compared with the SNAC-based reference product. **Results:** C10 exhibited sufficient buffering capacity to neutralize acidic environments. In Caco-2 and rat PK studies, C10 enhanced semaglutide absorption in a concentration-dependent manner, yielding exposure levels equivalent to SNAC at matched doses. Among the tablet designs, the monolayer tablet showed the highest dissolution similarity (*f*_2_ = 67.8) to Rybelsus. In beagle dogs, the optimized monolayer formulation produced pharmacokinetic parameters, including *C*_max_, *AUC*_last_, and *t*_1/2_, that overlapped with those of the SNAC-based reference drug product under matched dosing conditions. **Conclusions:** These results demonstrate that C10 can effectively support oral semaglutide delivery when incorporated into a rationally designed IR tablet. The findings support the feasibility of MCFA-based permeation enhancer platforms as formulation alternatives to SNAC for oral peptide therapeutics.

## 1. Introduction

Semaglutide is a long-acting glucagon-like peptide-1 receptor agonist (GLP-1 RA) that has demonstrated exceptional efficacy in managing type 2 diabetes and obesity, providing proven benefits in glycemic control, weight loss, and cardiovascular risk reduction [1,2,3,4]. These benefits led to the success of injectable semaglutide therapies (e.g., Ozempic and Wegovy) and established semaglutide as a cornerstone treatment in its class [3]. However, like most peptide drugs, semaglutide faces formidable barriers to oral delivery. It is highly susceptible to enzymatic degradation in the gastrointestinal (GI) tract and exhibits very low transepithelial permeability, resulting in an oral bioavailability typically below 1% [5,6,7,8]. Achieving clinically meaningful plasma levels via the oral route thus requires specialized formulation strategies to overcome proteolytic and absorption hurdles.

A major advancement in this arena was the introduction of oral semaglutide (Rybelsus), which co-formulates semaglutide with the absorption enhancer sodium *N*-(8-[2-hydroxybenzoyl]amino) caprylate (SNAC) [2]. SNAC is a synthetic derivative of caprylic acid that facilitates the uptake of semaglutide in the stomach through a unique mechanism: after tablet disintegration, a high local concentration of SNAC raises the gastric pH and increases the fluidity of the mucosal cell membranes without opening tight junctions, thereby enabling transcellular transport of the peptide across the gastric epithelium [2]. This SNAC-based technology enabled the first oral GLP-1 RA therapy and demonstrated the feasibility of oral delivery of a large peptide in humans [9].

Nevertheless, important limitations remain. SNAC-mediated absorption is inherently low in efficiency and highly variable [10]. Achieving even this level of exposure necessitates high doses of excipients and strict dosing requirements (e.g., prolonged fasting and minimal co-ingestion of liquids) to create the appropriate gastric environment [10]. Furthermore, the enhancement effect of SNAC is mechanism-specific and localized to the stomach, which may not readily translate to other peptides or less controlled GI conditions [11]. These formulation and performance constraints highlight the need to explore alternative permeation enhancers that could offer different or potentially complementary absorption-enhancing characteristics while maintaining safety [12].

Medium-chain fatty acids (MCFAs) have long been investigated as permeation enhancers, and sodium caprate (C10) is a well-established representative of this class [13]. In addition to its established role as a permeation enhancer, C10 is of translational interest because it is not a completely novel synthetic excipient from a regulatory perspective. Standard reference data indicate that C10 has an LD_50_ of 3.7 g/kg following oral gavage in rats [10,13]. Furthermore, C10 has been approved by the FDA as a direct food additive and, within the food sector, has been evaluated without a specified daily intake limit [14,15]. Mechanistically, C10 promotes macromolecular absorption by reversibly opening tight junctions and modulating cell membrane structure in the intestinal epithelium [10]. This differs from SNAC’s primarily transcellular, pH-dependent mode of action. C10 has been utilized in various oral delivery platforms and has demonstrated transient, reversible enhancement of peptide absorption in both preclinical models and clinical trials [10,11,14,16,17,18]. Notably, SNAC and C10 are two of the most advanced oral absorption enhancers tested in humans, each with a substantial track record of development. In vitro and in vivo comparisons suggest that neither agent holds a decisive advantage in efficacy: both typically yield only single-digit percentage increases in peptide bioavailability accompanied by considerable inter-subject variability [2]. Despite this, no systematic, formulation-integrated comparison of SNAC and C10 has been reported in the context of oral semaglutide. In other words, it remains unclear whether a C10-based immediate-release(IR) semaglutide tablet can achieve systemic exposure comparable to that of the SNAC-based reference formulation under equivalent conditions.

In addition to its established permeability-enhancing effects, we explored whether C10 could effectively function within the gastric environment of an oral semaglutide formulation. Although C10 is a weaker alkalizing agent than SNAC, its potential effectiveness is supported by the fact that the buffering capacity of the fasted human stomach is reported to be relatively low [19,20]. This inherent buffering capacity is expected to be similarly limited in beagle dogs, which exhibit lower basal acid secretion rates than humans [21,22]. Under such low-buffer conditions, the alkalizing potential of C10 may be sufficient to modulate the local microenvironmental pH, thereby attenuating pepsin activity and ensuring peptide stability. This rationale led us to investigate an IR tablet designed to achieve rapid co-localization of semaglutide and C10 in the stomach.

To address this knowledge gap, the present study employed a formulation-centric, stepwise development approach to evaluate C10 as a permeation enhancer for oral semaglutide [23]. Initial mechanistic studies, including Caco-2 permeation assays and rat pharmacokinetic evaluations, were conducted to establish the concentration-dependent absorption-enhancing potential of C10 relative to SNAC. Guided by these findings, IR tablet prototypes incorporating semaglutide and C10 were developed to promote rapid disintegration and to create local microenvironmental conditions favorable for enhancer action, particularly through pH modulation and efficient dispersion under gastric-relevant conditions. The resulting formulation designs were assessed via biorelevant dissolution testing, and the lead prototype was further evaluated in beagle dogs to characterize systemic exposure and translational pharmacokinetic performance. This integrated strategy enabled a side-by-side evaluation of C10-based and SNAC-based semaglutide formulations across preformulation, in vitro, and in vivo stages. Overall, the study aimed to determine whether a rationally designed C10-containing IR tablet could achieve systemic semaglutide exposure comparable to that of the clinically established SNAC-based reference, while providing an objective assessment of the feasibility and limitations of C10 as an alternative enhancer for oral peptide delivery.

## 2. Materials and Methods

### 2.1. Materials

Semaglutide (peptide purity > 98%) was obtained from Fujian Genohope Biotech Ltd. (Putian, China) and Sinopep-Allsino Biopharmaceutical Co., Ltd. (Hangzhou, China). Sodium *N*-[8-(2-hydroxybenzoyl)amino]caprylate (SNAC, purity > 99%) was purchased from GLPBIO (Montclair, CA, USA). Sodium caprate was purchased from Tokyo Chemical Industry Co., Ltd. (Tokyo, Japan). The reference drug product (Rybelsus^®^ 14 mg tablets) was manufactured by Novo Nordisk (Bagsværd, Denmark). Lactose monohydrate (Pharmatose 200 M) was obtained from DFE Pharma GmbH & Co. KG (Goch, Germany). Povidone K90 was obtained from Ashland Inc. (Wilmington, DE, USA). Microcrystalline cellulose (MCC, Heweten 102) was obtained from JRS Pharma (Rosenberg, Germany). Crospovidone (Kollidon CL) and copovidone (Kollidon VA64) were obtained from BASF (Ludwigshafen, Germany). Sodium starch glycolate was obtained from Roquette (Lestrem, France). Croscarmellose sodium (Ac-Di-Sol) was obtained from IFF (Wilmington, DE, USA). Sodium bicarbonate was obtained from Hebei Huachen Pharmaceutical Co., Ltd. (Cangzhou, China). Magnesium stearate was obtained from Nitika Pharmaceuticals Ltd. (Maharashtra, India). Hank’s balanced salt solution (HBSS) and 4-(2-hydroxyethyl)-1-piperazineethanesulfonic acid (HEPES) buffer solution were obtained from Welgene Inc. (Gyeongsan, Republic of Korea). Hydrochloric acid and Tween 80 were purchased from Daejung Chemicals & Metals Co., Ltd. (Siheung, Republic of Korea). MTT reagent, 3-(4,5-dimethylthiazol-2-yl)-2,5-diphenyltetrazolium bromide, was purchased from Sigma-Aldrich (St. Louis, MO, USA). The LDH cytotoxicity assay was performed using a Cytotoxicity LDH Assay Kit-WST purchased from Dojindo Molecular Technologies, Inc. (Kumamoto, Japan). High-performance liquid chromatography (HPLC)-grade acetonitrile and methanol were obtained from Duksan Pure Chemicals Co., Ltd. (Ansan, Republic of Korea). All other chemicals and solvents were of analytical grade and were used as received. Ultrapure water (18.2 MΩ·cm) was prepared using an in-house purification system.

### 2.2. Preformulation Studies

The preformulation strategy for the present study was established to support the rational design of oral semaglutide tablets. While semaglutide solubility and excipient compatibility were previously characterized in our quality-by-design (QbD)-based study [24], the current work focused on further characterizing the morphology of semaglutide raw materials and the acid-neutralizing capacity and dispersion behavior of C10. These assessments were critical to refining the selection of C10-containing IR tablet architectures.

#### 2.2.1. Morphology

The morphology of semaglutide was examined using an optical microscope (IX2-ILL100; Olympus, Tokyo, Japan) to evaluate three distinct material profiles: two different batches from one supplier (Genohope 1 and 2) and one from an alternative supplier (Sinopep, Hangzhou, China). Samples were dispersed on glass slides, and representative images were captured at 100× and 400× magnification in bright-field mode. The particle size distribution, degree of agglomeration, and overall physical uniformity were qualitatively assessed to evaluate lot-to-lot and supplier-dependent variability, as well as their potential impact on blend uniformity and downstream formulation performance.

#### 2.2.2. Preliminary Assessment of Acid-Neutralization and Dispersion Behavior of C10 Under Gastric-Relevant Conditions

To evaluate the acid-neutralizing and dispersion behavior of C10, conditions reflecting fasted gastric fluid volumes in beagle dogs and humans were established. The canine-mimetic condition consisted of 25 mL of 0.01 N HCl supplemented with 10 mL of water, while the human-mimetic condition consisted of 50 mL of 0.01 N HCl supplemented with 120 mL of water [25,26,27,28,29]. Each medium was prepared in a 200 mL beaker, and C10 was added at 100, 300, or 500 mg at room temperature. The mixtures were stirred with a magnetic stir bar at 150 rpm for 15 min. The pH was measured immediately after the 15 min stirring period and again after an additional 30 min using a pH meter (Starter 3100; OHAUS Corporation, Parsippany, NJ, USA).

Dispersion properties were evaluated using a dynamic light scattering (DLS) particle size analyzer (ELSZ-2000, Otsuka Electronics Co., Ltd., Osaka, Japan). Approximately 3.0 mL of each prepared sample was transferred into a disposable polymethyl methacrylate (PMMA) cuvette, and measurements were performed at 25 °C. The *Z*-average diameter, particle size distribution, and polydispersity index (PDI) were determined. Each DLS measurement was performed with 50 accumulations and repeated six times for each sample. The resulting data were analyzed using the instrument software to characterize the particle size properties of the C10 dispersion system.

### 2.3. Formulation of Semaglutide Oral Tablets

A dry granulation-based manufacturing process was established to ensure the physicochemical stability of semaglutide, which is susceptible to moisture and heat, while simultaneously achieving excellent flowability and compressibility. The development of these IR oral tablets was further guided by preformulation findings, excipient compatibility results, and the functional role of C10 as a medium-chain fatty acid (MCFA)-derived permeation enhancer. Three tablet architectures—single-layer (SSL-T), bilayer (SBL-T), and dry compression-coated (SCC-T) tablets—were engineered to systematically evaluate the influence of enhancer placement, microenvironmental pH, and compression configuration on semaglutide release and absorption [30,31].

#### 2.3.1. Preparation of Single-Layer Tablets (SSL-Ts)

For the preparation of SSL-T, semaglutide (14.0 mg), sodium caprate (300.0 mg), lactose monohydrate (300.0 mg), croscarmellose sodium (17.0 mg), and copovidone (13.0 mg) were accurately weighed and manually blended for approximately 100 cycles to ensure a uniform distribution of the drug and the enhancer (Table 1). The blended mixture was then subjected to dry granulation via slugging. Slugging was performed using a rotary tablet press (PR-LM 08; PTK Co., Ltd., Gimpo-si, Republic of Korea) equipped with 15.0 mm round punches and dies under a compression force of 20–25 kN. The resulting slugs were milled using a hammer mill (Polymix PX-MFC 90 D; Kinematica AG, Malters, Switzerland) and passed through a 3.0 mm sieve to obtain free-flowing granules. Subsequently, magnesium stearate (8.0 mg) was incorporated as a lubricant with 50 mixing cycles. Finally, the granules were compressed into tablets using the same rotary tablet press equipped with 16.7 × 8.3 mm oblong punches. The pre-compression force was set at ≤1.0 kN, and the main compression force was maintained at ≥6.6 kN to ensure the mechanical strength and rapid disintegration required for IR tablets.

#### 2.3.2. Preparation of Bilayer Tablets (SBL-Ts)

SBL-T was designed to separate semaglutide from C10 during initial dissolution (Table 2). The tablet comprised two distinct layers: (i) an upper layer containing semaglutide (14.0 mg), lactose monohydrate (70.0 mg), microcrystalline cellulose (100.0 mg), and povidone K90 (5.0 mg); and (ii) a lower layer composed of sodium caprate (300.0 mg), lactose monohydrate (200.0 mg), crospovidone (30.4 mg), and copovidone VA64 (12.6 mg).

Each layer was granulated separately via the same slugging and milling procedure described for SSL-T, sieved through a 3.0 mm mesh, and lubricated with magnesium stearate (2.0 mg for the upper layer and 5.5 mg for the lower layer) with 50 mixing cycles. The bilayer tablets were manufactured using a bilayer rotary compression machine (PR-3000 Series; PTK Co., Ltd., Gimpo-si, Republic of Korea) equipped with 15.6 × 7.8 mm oblong punches.

The manufacturing process involved a sequential layering technique: the lower-layer granules (C10 layer) were first filled into the die and lightly pre-tamped, followed by the addition of the upper-layer granules (drug layer). The pre-compression force was set at ≤1.0 kN, and the final tablets were compressed under a main compression force of 13–14 kN. This compression profile was maintained to ensure adequate interlayer adhesion and to prevent physical defects such as capping or layer separation.

#### 2.3.3. Preparation of Dry Compression-Coated Tablets (SCC-Ts)

SCC-T was designed as a core–shell system to control the timing of semaglutide exposure to C10 (Table 3). The tablet comprised (i) an inner core containing semaglutide (14.0 mg), microcrystalline cellulose (77.0 mg), crospovidone (5.0 mg), and povidone K90 (2.0 mg); and (ii) an outer layer composed of C10 (300.0 mg), lactose monohydrate (330.0 mg), crospovidone (30.4 mg), and copovidone VA64 (12.6 mg).

Each component for the core and outer layers was dry-granulated separately via the same slugging and milling procedure described for SSL-T, sieved through a 3.0 mm mesh, and subsequently lubricated with magnesium stearate (2.0 mg for the core and 7.0 mg for the outer layer) with 50 mixing cycles.

The lubricated core granules were compressed into core tablets using a rotary tablet press equipped with 6.0 mm round punches under a compression force of ≥0.5 kN. For compression coating, the outer layer granules were first filled into a die, and the core tablet was centrally positioned. The final compression-coated tablets were then produced using a rotary compression machine equipped with 12.5 mm round punches under a compression force of 5–6 kN.

This core–shell architecture enabled evaluation of how delayed exposure of semaglutide to C10 influences dissolution behavior and in vivo absorption [32].

### 2.4. In Vitro Study

In vitro experiments were performed to characterize semaglutide dissolution from test formulations and to evaluate the permeability-enhancing effects of C10 using Caco-2 monolayers. Dissolution testing was performed under physiologically relevant pH conditions to simulate gastric and intestinal environments. Caco-2 transport studies assess apical-to-basolateral semaglutide transport and epithelial tolerance during enhancer exposure. All analyses were performed using validated analytical methods.

#### 2.4.1. In Vitro Dissolution Test

The dissolution behavior of semaglutide was assessed using the reference drug product and three test formulations (SSL-T, SBL-T, and SCC-T). Dissolution testing was performed using a dissolution tester (708-DS, Agilent Technologies, Santa Clara, CA, USA) in accordance with USP <711> using Apparatus II (paddle method). Three dissolution media (900 mL each) were used to reflect physiologically relevant gastrointestinal pH conditions: pH 1.2 simulated gastric fluid containing 0.75% Brij 35, pH 4.0 acetate buffer containing 0.75% Brij 35, and pH 6.8 phosphate buffer. To improve semaglutide solubility under acidic conditions and maintain sink conditions, Brij 35 was included in the pH 1.2 and pH 4.0 media. Dissolution testing was conducted at 37.0 ± 0.5 °C with a paddle rotation speed of 50 rpm.

For each test condition, six tablets (*n* = 6) were evaluated, and 4 mL samples were withdrawn at 5, 10, 15, 20, 30, 45, and 60 min. The collected samples were immediately filtered through 0.45 µm PTFE syringe filters and analyzed by HPLC using an Agilent 1260 Infinity II system (Agilent Technologies, Santa Clara, CA, USA) equipped with an Agilent C18 column (4.6 × 150 mm, 5 µm). The flow rate was 1.0 mL/min, the injection volume was 40 µL, and detection was performed at 220 nm. Dissolution profile similarity between the test and reference products was assessed using the similarity factor (*f*_2_). For the *f*_2_ calculation, different time points were selected depending on the dissolution behavior in each medium. Specifically, under the pH 1.2 condition, five time points (10, 15, 30, 45, and 60 min) were included because the reference product exceeded 85% dissolution at 60 min, whereas under the pH 4.0 and pH 6.8 conditions, three time points were used for each medium (pH 4.0: 10, 20, and 30 min; pH 6.8: 10, 15, and 30 min). An *f*_2_ value of 50 or greater was considered to indicate similarity between the two dissolution profiles.

#### 2.4.2. Caco-2 Cell Assay for In Vitro Permeability

Differentiated Caco-2 monolayers grown on 12 mm Transwell^®^ inserts with a 0.4 µm PET membrane (Corning, NY, USA) were used to evaluate the in vitro permeability of semaglutide. The apical transport buffer consisted of Hank’s balanced salt solution (HBSS) supplemented with 25 mM HEPES (pH 7.4), whereas the basolateral transport buffer consisted of HBSS supplemented with 25 mM HEPES (pH 7.4) and 0.02% (*w*/*v*) Tween 80 to minimize nonspecific adsorption of the peptide [33,34]. Prior to the transport experiment, monolayer integrity was confirmed by measuring transepithelial electrical resistance (TEER), and only inserts meeting the predefined acceptance criteria were used. The monolayers were washed twice with pre-warmed transport buffer in both the apical and basolateral chambers and equilibrated at 37 °C for 15 min.

Semaglutide was prepared at a final concentration of 200 µM and added to the apical chamber either alone or in combination with permeation enhancers. Based on the experimental design, C10 was tested at 2.5, 5, and 10 mM, whereas SNAC was tested at 10 and 20 mM. The apical and basolateral chambers received 0.5 mL of sample solution and 1.5 mL of transport buffer, respectively. The plates were then incubated at 37 °C in a 5% CO_2_ atmosphere for 1 h, after which samples were collected from both apical and basolateral compartments for quantitative analysis. Apparent permeability coefficients (*P_app_*) were calculated according to the following equation:

$$P_{app}=\frac{dQ}{\left(A\times C_{0}\times dt\right)}$$

where dQ/*dt* is the steady-state flux (µM/s) of semaglutide across the monolayer, *A* is the membrane surface area (cm^2^), and *C*_0_ is the initial semaglutide concentration in the apical chamber (µM).

Collected transport samples were quantified by RP-HPLC using a Gemini C18 column (150 × 4.6 mm, 5 µm; Phenomenex, Torrance, CA, USA). Chromatographic analysis was performed at 30 °C with a flow rate of 1.0 mL/min. The mobile phase consisted of (A) 0.1% trifluoroacetic acid (TFA) in DW and (B) 0.1% TFA in acetonitrile (ACN), with the following gradient program: 40% B at 0–0.5 min, 70% B at 10.0–11.0 min, and 40% B at 11.5–15.0 min. Semaglutide was detected by UV absorbance at 280 nm and fluorescence detection at λ_ex_ 280 nm and λ_em_ 340 nm. In parallel, 3-(4,5-dimethylthiazol-2-yl)-2,5-diphenyltetrazolium bromide (MTT) and Lactate dehydrogenase (LDH) assays were performed to assess potential cytotoxicity and barrier disruption associated with enhancer exposure.

### 2.5. In Vivo Oral Permeability Study

In vivo studies were conducted in Sprague–Dawley (SD) rats and beagle dogs to characterize the pharmacokinetic (PK) profiles of semaglutide following oral administration of formulations containing the MCFA-derived permeation enhancer C10 or SNAC, and the reference drug product. All animal experiments were conducted in accordance with institutional and national guidelines for the care and use of laboratory animals and received approval from the relevant Institutional Animal Care and Use Committees (IACUCs).

#### 2.5.1. Pharmacokinetic Evaluation of Oral Semaglutide with C10 in Sprague–Dawley Rats

Male SD rats (7 weeks old, 230–270 g) were obtained from Young Bio (Seongnam, Gyeonggi, Republic of Korea). Animals were acclimatized for a minimum of one week under controlled environmental conditions (temperature 21 ± 2 °C, relative humidity 35–65%, 12 h light/dark cycle, and 10–15 air changes per hour) with free access to standard chow and water. The study protocol received approval from the IACUC of Dongduk Women’s University (approval no. 202407-01, on 18 July 2024).

Prior to dosing, the rats were fasted for approximately 16 h with free access to water. Animals were randomly allocated to six treatment groups (*n* = 5 per group) and administered the following oral treatments: Group 1, distilled water (vehicle control); Group 2, semaglutide (3 mg/kg); Group 3, semaglutide (3 mg/kg) with C10 (100 mg/kg); Group 4, semaglutide (3 mg/kg) with C10 (200 mg/kg); Group 5, semaglutide (3 mg/kg) with C10 (300 mg/kg); and Group 6, semaglutide (3 mg/kg) with SNAC (200 mg/kg). Semaglutide and the enhancers were suspended in an appropriate aqueous vehicle immediately before administration and administered by oral gavage at a dose volume adjusted according to body weight.

Blood samples (approximately 350 µL per time point) were collected from the jugular vein into dipotassium ethylenediaminetetraacetate (K_2_-EDTA) vacuatainer tubes at predetermined intervals (pre-dose and 0.5, 1, 2, 4, 6, 8, and 24 h post-dose). Samples were centrifuged at 12,000 rpm for 5 min at 4 °C to obtain plasma according to the rate study-specific plasma preparation procedure. The supernatant was transferred into labeled tubes and stored at −70 °C until quantitative analysis of semaglutide was performed using a validated liquid chromatography–tandem mass spectrometry (LC–MS/MS, QTRAP^®^ 6500, AB Sciex LLC, Framingham, MA, USA) method.

#### 2.5.2. Pharmacokinetic Evaluation of Semaglutide After Oral Administration in Beagle Dogs

PK studies were conducted in beagle dogs to compare the systemic exposure to semaglutide following oral administration of the reference drug product and the optimized semaglutide tablet formulation. Male beagle dogs (2–3 years old, 10–12 kg) were obtained from Raonbio Inc. (Yongin, Gyeonggi, Republic of Korea). Animals were individually housed in stainless-steel cages under controlled environmental conditions (temperature 21 ± 2 °C, relative humidity 35–65%, 12 h light/dark cycle, and 10–15 air changes per hour). During the acclimatization period, animals were provided with free access to water and a standard canine diet. All experimental procedures were conducted in accordance with institutional animal welfare policies and national regulations governing the care and use of laboratory animals. The study protocol was reviewed and approved by the IACUC of the NDIC (approval no. NDIC-IACUC P243036, approved on 16 August 2024).

Before administration, the dogs were fasted for approximately 16 h, with water provided ad libitum. Animals were randomly assigned to two treatment groups and were administered a single oral dose. The reference group received a commercial semaglutide tablet (Rybelsus 14 mg, *n* = 5), and the test group received the SSL-T (14 mg, *n* = 4). Each tablet was administered with approximately 20 mL of water to ensure complete swallowing, and food was reintroduced 4 h post-dosing.

Blood samples (approximately 3 mL per time point) were collected from the jugular vein into K_2_-EDTA vacutainer tubes at pre-dose (0 h) and at 0.25 h, 0.5 h, 1 h, 2 h, 3 h, 4 h, 6 h, 8 h, 12 h, 24 h, and 48 h following administration. Immediately after collection, the samples were gently inverted to ensure anticoagulation and subsequently centrifuged at 4000 rpm for 10 min at 4 °C according to the beagle dog study-specific plasma preparation procedure. Plasma was separated subsequently and stored at −70 °C until semaglutide concentrations were quantified using LC–MS/MS.

### 2.6. Liquid Chromatography–Tandem Mass Spectrometry Quantitative Analysis

Plasma concentrations of semaglutide in rat and beagle dog studies were quantified using a validated LC–MS/MS (QTRAP^®^ 6500, AB Sciex LLC, Framingham, MA, USA) method. Sample preparation was conducted using protein precipitation. Briefly, 50 µL of plasma was aliquoted into 1.5 mL microtubes and mixed with 50 µL of an internal standard solution (liraglutide, 100–300 ng/mL in acetonitrile), followed by the addition of 200 µL of methanol. The mixture was vortexed for 5 min and subsequently centrifuged at 17,600× *g* for 5 min at 4 °C. The supernatant (150 µL) was transferred into autosampler vials, and 10 µL was injected into the LC–MS/MS system for analysis.

Chromatographic separation was performed using an Agilent 1290 Infinity II system equipped with a Waters ACQUITY UPLC^®^ CSH^TM^ C18 column (2.1 × 50 mm, 1.7 µm). The mobile phases comprised (A) water with 0.1% formic acid and (B) acetonitrile with 0.1% formic acid. A linear gradient was employed as follows: 35% B at 0.0–0.25 min, 75% B at 3.0 min, 90% B at 3.10–3.20 min, and 35% B at 3.40–4.00 min. The flow rate was 0.3 mL/min, increased to 0.4 mL/min after 3.10 min, and was maintained until 4.00 min. The column temperature was maintained at 40–50 °C, and the autosampler was kept at 10 °C.

Mass spectrometric detection was performed using a SCIEX QTRAP 6500 system equipped with an electrospray ionization source operating in positive ion mode. Quantification was conducted using multiple-reaction monitoring (MRM). The optimized MRM transitions were *m*/*z* 1029.167 → 1238.000 for semaglutide and *m*/*z* 938.608 → 1064.000 for the internal standard (liraglutide). Instrument parameters were set as follows: the curtain gas was 30 psi, ion source gases 1 and 2 were set at 50 psi, ion spray voltage was 5500 V, and source temperature was 400 °C.

Calibration standards were prepared through spiking blank plasma to yield final semaglutide concentrations of 1, 5, 10, 50, 100, 500, and 1000 ng/mL. Linearity was assessed using weighted (1/*x*^2^) linear regression, and correlation coefficients (r ≥ 0.99) were considered acceptable. The lower limit of quantification (LLOQ) was 1 ng/mL, which was defined by a signal-to-noise ratio of ≥ 5 and accuracy and precision within ±20%. Quality-control samples at low, medium, and high concentrations were included to verify the accuracy and precision based on FDA/EMA bioanalytical guidelines.

Chromatograms were processed using Analyst 1.6.3 software, and semaglutide concentrations were determined based on the ratios of analyte-to-internal-standard peak-area ratios. Concentrations below the LLOQ were reported as below the quantification limit (BQL). All validated analytical results were subsequently employed for PK calculations (Section 2.7).

### 2.7. Pharmacokinetic Analysis

PK parameters were calculated using standard non-compartmental analysis (NCA) with Phoenix^®^ WinNonlin^®^ software version 8.3.2 (Pharsight, Mountain View, CA, USA). The area under the plasma concentration–time curve from time zero to the last quantifiable concentration (*AUC*_last_) was determined using the linear-up/log-down trapezoidal method. The area under the curve extrapolated to infinity (*AUC*_inf_) was determined as *AUC*_last_ + *C*_last_/*k*_el_, where *k*_el_ was the terminal elimination rate constant obtained from the log-linear regression of the terminal phase.

The terminal half-life (*t*_½_) was calculated as 0.693/*k*_el_. The maximum plasma concentration (*C*_max_) and the time to reach this concentration (*t*_max_) were determined directly from the observed individual concentration–time profiles. All PK parameters were calculated using actual sampling times, and values BQL were treated as zero prior to the first quantifiable time point and as missing thereafter [35].

### 2.8. Statistical Analysis

Data were expressed as means ± *SD*. Treatment means were compared by one-way ANOVA, and pairwise comparisons were calculated using the least significant difference (LSD) test. Differences were considered statistically significant when *p* ≤ 0.05. Minitab^®^ version 21.0 (Minitab Inc., University Park, PA, USA) was used for all statistical analyses.

## 3. Results

### 3.1. Preformulation Characteristics Relevant to Formulation Design (Morphology, Acid-Neutralizing, and Dispersion Behavior of C10)

Preformulation evaluation of the physicochemical and biopharmaceutical properties of the active pharmaceutical ingredient is required for the development of oral semaglutide formulations. The formulation basis for the present study was established in our previously published QbD-based study [24], in which semaglutide solubility characteristics and excipient compatibility were evaluated to provide essential information for initial formulation design. Specifically, the dissolution behavior of semaglutide under various pH conditions was assessed to predict its behavior in the gastrointestinal environment, and compatibility between the drug substance and candidate excipients was examined to identify excipients suitable for formulation development. Building on these findings, the present study further evaluated the morphology of semaglutide and the acid-neutralizing and dispersion behavior of C10, thereby refining material selection and providing additional rationale for the design of C10-containing IR tablets.

#### 3.1.1. Morphology

**Figure 1:**
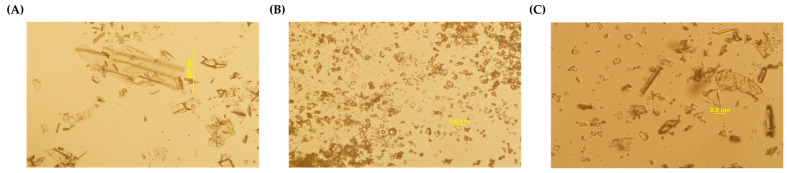
Microscopic morphology of semaglutide particles for three drug commercial manufacturers: (**A**) Genohope 1 (100×), (**B**) Genohope 2 (400×), and (**C**) Sinopep (400×).

As exhibited in Figure 1, optical microscopy (OM, OLYMPUS IX2-ILL100, Olympus Corporation, Japan) revealed distinct differences in the particle size and morphological distribution among the three semaglutide drug substances. Genohope 1 exhibited relatively large particles, ranging from approximately 10–20 µm, with an irregular distribution and noticeable agglomeration. In contrast, Genohope 2 exhibited a markedly smaller mean particle size of less than 2 µm, with a highly uniform and fine distribution. Sinopep exhibited an intermediate profile, demonstrating a consistent particle size range of 2–6 µm and maintaining a compact, stable morphology.

These differences were likely attributable to the respective manufacturing processes—including milling and granulation—and were expected to influence downstream formulation performance. In particular, the tighter and more uniform particle distribution of Sinopep indicated advantages in blend uniformity and content homogeneity during tablet manufacturing. Moreover, variation in particle size among the three materials may exert consequential effects on the dissolution rate and physicochemical stability (Figure 1). Based on these morphology results and material availability, the Sinopep semaglutide drug substance was selected for subsequent tablet formulation, in vitro dissolution testing, Caco-2 permeability studies, and in vivo pharmacokinetic evaluations.

#### 3.1.2. Acid-Neutralizing and Dispersion Behavior of C10 Under Gastric-Relevant Conditions

To achieve successful oral delivery of semaglutide, overcoming the harsh acidic environment of the stomach and ensuring the functional state of the absorption enhancer are paramount. In this context, we evaluated the acid-neutralizing capacity and subsequent dispersion behavior of C10 under simulated gastric conditions for both beagle dogs and humans, as these parameters directly govern the solubility and permeation-enhancing efficiency of the system.

As demonstrated in Table 4, C10 exhibited sufficient neutralizing capacity and a dose-dependent buffering effect. In the simulated beagle gastric condition, C10 effectively neutralized the 0.01 N HCl medium, maintaining a stable pH range (6.32–7.63) across all tested doses (100–500 mg). This suggests that even a minimal dose of C10 can provide a favorable local pH for drug dissolution in the canine gastric environment. Conversely, under simulated human gastric conditions, the 100 mg dose failed to fully shift the pH out of the acidic range (pH 4.98), whereas doses of 300 mg and 500 mg successfully established a neutral environment (pH > 6.2). These results highlight that a threshold dose of C10 is required to bypass the pH-dependent solubility barrier in humans.

Furthermore, the physical integrity of the C10 dispersion was scrutinized via DLS analysis to ensure its readiness for membrane interaction. As summarized in Table 5, the 300 mg dose in both gastric models maintained a relatively narrow particle size distribution, evidenced by low PDI values (0.29 and 0.19 for beagle and human conditions, respectively) and consistent *Z*-average diameters, which indicates the formation of a stable and uniform micellar system. However, under human conditions, the 500 mg dose led to large-scale aggregation, with the *Z*-average diameter reaching 17,642.2 nm and the *D*_90_ value exceeding 120,000 nm. This drastic increase in both the *Z*-average and the polydispersity of the system suggests a collapse of the fine dispersion, resulting in highly heterogeneous aggregates. Overall, these preliminary assessments identify 300 mg as the optimal dose that simultaneously satisfies both the requirement for acid neutralization and the maintenance of a favorable dispersion state for oral absorption.

### 3.2. In Vitro Study

#### 3.2.1. In Vitro Dissolution Test

The dissolution behavior of semaglutide was assessed using the reference drug product and three test formulations (SSL-T, SBL-T, SCC-T). Dissolution testing was conducted in accordance with USP <711> using Apparatus II (paddle method) at 37 ± 0.5 °C in pH 1.2 (with 0.75% Brij 35), pH 4.0 (with 0.75% Brij 35), and pH 6.8 phosphate buffer. Samples were collected using a dissolution tester (Agilent 708-DS, Agilent Technologies, Santa Clara, CA, USA) for up to 120 min and subsequently analyzed using HPLC.

**Figure 2:**
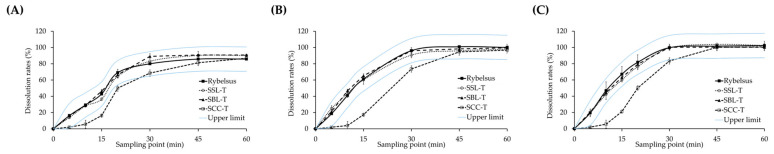
Dissolution profiles of the reference drug product and Semaglutide formulations (SSL-T, SBL-T, and SCC-T) in (**A**) pH 1.2 medium containing 0.75% Brij 35, (**B**) pH 4.0 medium containing 0.75% Brij 35, and (**C**) pH 6.8 phosphate buffer at 37 ± 0.5 °C using USP Apparatus II (paddle method).

As exemplified in Figure 2, SSL-T and SBL-T exhibited rapid dissolution, with more than 80% of the drug release within 45 min across all media, which was consistent with the characteristics of IR formulations. Their dissolution profiles closely aligned with those of the reference drug product (Rybelsus). The similarity factor analysis further confirmed this observation: SSL-T exhibited an *f*_2_ value of 67.8 (10–60 min, five time points), and SBL-T exhibited an *f*_2_ value of 64.6 (10–45 min, four time points). The values exceeded the threshold of 50, indicating dissolution similarity to the reference drug product.

In contrast, SCC-T exhibited an initial lag phase of approximately 10 min, due to the disintegration of the outer coat prior to the exposure of the drug-containing core. Following this lag period, the drug release increased rapidly; however, the similarity factor (*f*_2_ = 36.2, 10–60 min, five time points) remained below 50, indicating dissimilarity relative to the reference drug product. Although the later-phase dissolution of SCC-T approached that of the reference drug product, the initial delay in release contributed to the reduced similarity value (Figure 2).

Overall, SSL-T and SBL-T demonstrated dissolution behaviors equivalent to those of the reference drug product based on release behavior and *f*_2_ similarity factors. In contrast, SCC-T showed a short initial delay that resulted in a non-similar *f*_2_ value despite ultimately achieving a comparable extent of dissolution. The selected time windows for *f*_2_ calculation were chosen according to regulatory guidance for IR products, focusing on early- and mid-phase dissolution behavior.

#### 3.2.2. Caco-2 Cell Assay for Oral Permeability

The oral absorption of semaglutide is primarily limited by its large molecular size and low intrinsic permeability across the intestinal epithelium. To address these barriers, we evaluated the performance of C10 as a permeation enhancer using Caco-2 cell monolayers. Prior to the transport studies, the establishment of a robust epithelial barrier was confirmed by TEER values (≥300 Ω·cm^2^), ensuring that the system reliably mimicked the physiological intestinal environment.

A critical step in this evaluation was determining the optimal balance between enhancement efficiency and cellular safety. Through preliminary MTT and LDH screening (Figure S1), we identified the concentration-dependent cytotoxic profile of each enhancer. While C10 at 10 mM reached a threshold of mild metabolic stress, the absence of significant LDH leakage indicated that the cell membrane’s physical continuity remained intact. Based on this rational selection process, we selected specific concentrations—2.5, 5, and 10 mM for C10 and 10 and 20 mM for SNAC—to compare their respective effects on semaglutide permeation.

**Figure 3:**
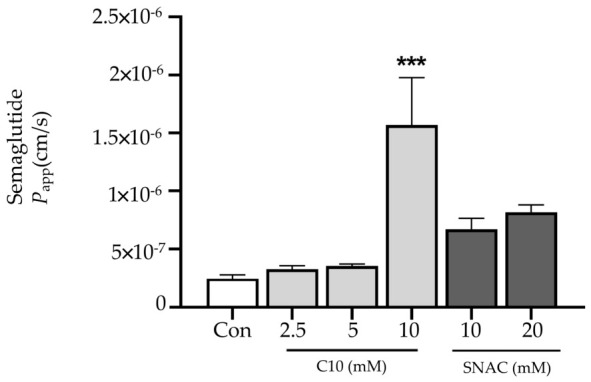
Concentration-dependent permeability enhancement of semaglutide using C10 and SNAC in Caco-2 monolayers. *** *p* < 0.001.

The quantitative results, as detailed in Table 6 and Figure 3, highlight the effectiveness of C10. Semaglutide alone exhibited a baseline *P_app_* of 2.49 × 10^−7^ cm/s, which was improved upon the addition of enhancers. Notably, C10 at 10 mM achieved a *P_app_* of 1.57 × 10^−6^ cm/s, representing a 6.30-fold increase relative to the control (*p* < 0.001). This level of enhancement was higher than that achieved by SNAC at 20 mM (3.28-fold increase). The fact that C10 at 10 mM provided a higher permeation flux compared to SNAC at a higher molar concentration (20 mM) demonstrates its distinct efficiency as a permeation-enhancing agent. These findings provided the initial mechanistic rationale to proceed with rat pharmacokinetic studies to further characterize the absorption-enhancing potential of C10 in a more complex biological environment.

### 3.3. In Vivo Evaluation

#### 3.3.1. Pharmacokinetic Evaluation of Oral Semaglutide in Sprague–Dawley Rats

**Figure 4:**
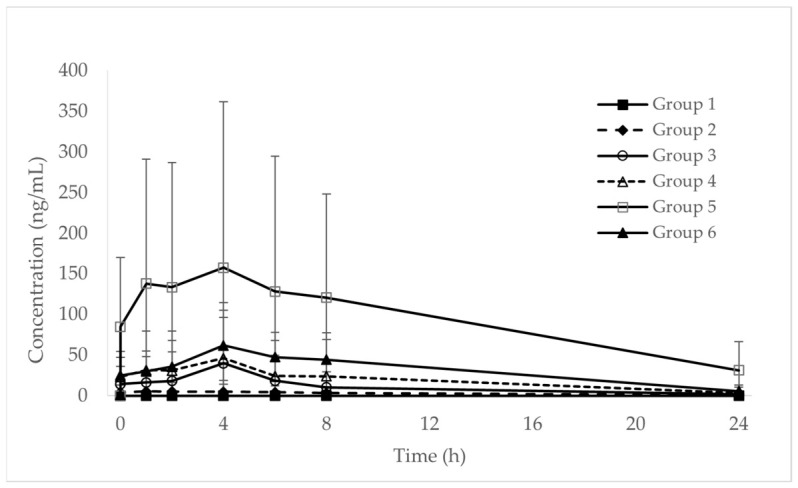
Plasma concentration–time profiles of semaglutide following oral administration of various formulations in Sprague–Dawley rats (*n* = 5).

To characterize the absorption-enhancing potential of C10 in a systemic environment, the PK profiles of semaglutide were analyzed following a single oral administration (3 mg/kg) in male SD rats. The plasma concentrations were quantified using a validated LC-MS/MS method, and the calculated PK parameters and mean plasma concentration–time profiles are summarized in Table 7 and Figure 4, respectively.

As anticipated, no semaglutide was detected in the negative control group (G1, DW). In the semaglutide-only group (G2), a baseline *C*_max_ of 9.92 ± 13.42 ng/mL was observed at a *t*_max_ of 3.00 h, with an *AUC*_last_ of 58.88 ± 76.38 ng·h/mL. These results confirm the poor intrinsic oral bioavailability of semaglutide in the absence of a permeation enhancer.

Upon co-administration with C10 (G3–G5), a clear dose-dependent increase in systemic exposure was observed. While G3 (C10 100 mg/kg) and G4 (C10 200 mg/kg) showed incremental improvements in absorption, G5 (C10 300 mg/kg) demonstrated a significantly more pronounced enhancement. Specifically, G5 achieved a *C*_max_ of 171.15 ± 201.29 ng/mL and an *AUC*_last_ of 1966.51 ± 2507.23 ng·h/mL, representing an approximate 33.4-fold increase in *AUC*_last_ compared to G2 (*p* < 0.001). This suggests that a dosage of 300 mg/kg effectively reaches the concentration threshold required to overcome the intestinal absorption barrier for semaglutide.

In comparison, the SNAC 200 mg/kg group (G6) exhibited an *AUC*_last_ of 503.09 ± 586.33 ng·h/mL. Although G6 showed slightly higher exposure than the equivalent dose of C10 (G4, 346.90 ± 719.77 ng·h/mL), the notably higher exposure achieved in G5 (300 mg/kg) indicates that C10 can provide superior absorption enhancement when optimized at this dose level. All formulations followed mono-exponential elimination kinetics with no observed clinical abnormalities, indicating that the evaluated doses were well tolerated. Considerable inter-animal variability was observed across the rat treatment groups, which is consistent with the variable nature of oral semaglutide absorption using permeation enhancers. Collectively, these findings validate the dose-responsive effects of C10 and provide a mechanistic rationale for incorporating C10 at a target dose of 300 mg in subsequent IR tablet formulations to replicate this effective high-concentration microenvironment.

#### 3.3.2. Pharmacokinetic Profiles of Semaglutide After Oral Administration in Beagle Dogs

**Figure 5:**
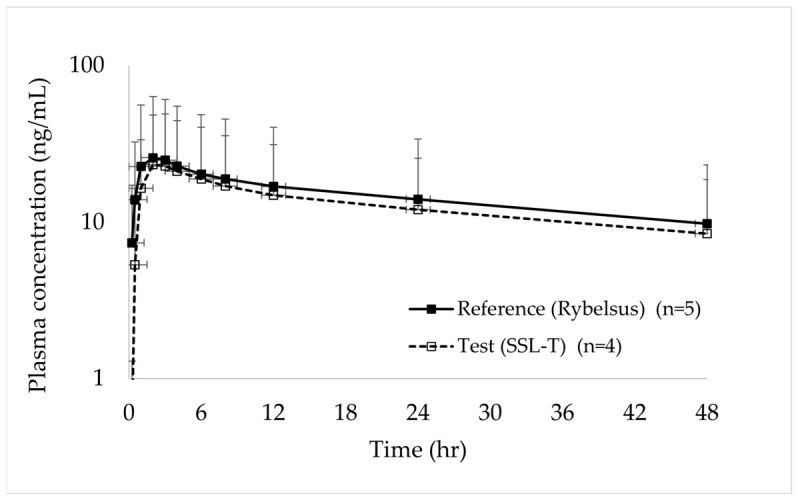
Plasma concentration–time profiles of semaglutide after oral administration of the reference drug product or SSL-T in beagle dogs. Plasma semaglutide concentrations were determined using LC–MS/MS.

The PK characteristics of semaglutide were further evaluated in beagle dogs following the oral administration of either the reference product (Rybelsus) or the SSL-T formulation (Figure 5; Table 8). While rodents typically show relatively high oral bioavailability for GLP-1 RAs, larger animals like beagle dogs often exhibit lower and more variable absorption, presenting a more rigorous challenge for oral delivery systems.

As demonstrated in Figure 5, the plasma concentration–time profiles for both groups were characterized by a rapid absorption phase within the first few hours, followed by a gradual mono-exponential decline over 48 h. The PK parameters, summarized in Table 8, revealed that systemic exposure was highly comparable between the two treatments. The reference product reached a *C*_max_ of 26.13 ± 37.56 ng/mL at a *t*_max_ of 3.0 ± 1.0 h, while SSL-T achieved a *C*_max_ of 23.84 ± 25.65 ng/mL with a slightly earlier *t*_max_ of 2.0 ± 0.82 h.

Estimates of overall exposure also demonstrated a high degree of similarity. The *AUC*_last_ values were 712.03 ± 995.25 ng·h/mL for the reference product and 619.96 ± 697.68 ng·h/mL for SSL-T. Furthermore, the terminal half-lives (*t*_1/2_) were nearly identical (42.30 ± 24.82 h for the reference and 42.32 ± 8.28 h for SSL-T), indicating consistent elimination kinetics between the SNAC-based and C10-based systems.

No abnormal clinical signs were observed during the study, confirming that the C10-based formulation was well tolerated at the evaluated dose. Collectively, these findings indicate that the SSL-T formulation provides systemic exposure and disposition characteristics functionally equivalent to those of the reference drug product. The attainment of reference-level exposure in a large animal model validates the effectiveness of our C10-based IR strategy, demonstrating that rationally designed C10-containing tablets can achieve performance comparable to that of established SNAC-based technologies.

## 4. Discussion

In this study, C10, an MCFA-derived permeation modulator, was evaluated as an alternative permeation enhancer for oral semaglutide delivery through an integrated assessment encompassing formulation design, dissolution behavior, epithelial permeability, and in vivo pharmacokinetics in both rats and beagle dogs. The overall findings support the feasibility of C10-based IR semaglutide tablet formulations as an alternative to SNAC-based technology and provide mechanistic and translationally relevant insights for the oral delivery of peptide therapeutics for obesity and diabetes.

### 4.1. Mechanistic Interpretation of Oral Absorption Enhancer-Mediated Permeability

Semaglutide exhibits intrinsically low epithelial permeability due to its unique physicochemical properties, such as high molecular weight and hydrophilicity, necessitating the use of permeation enhancers to achieve measurable systemic exposure following oral administration. In Caco-2 monolayers, C10 increased transepithelial transport of semaglutide in a concentration-dependent manner, resulting in a pronounced increase in apparent permeability compared with SNAC under the tested in vitro conditions. This observation is consistent with established MCFA-mediated mechanisms involving reversible modulation of membrane organization and transient loosening of tight junctions, which differ from the primarily transcellular and pH-dependent mechanism reported for SNAC.

### 4.2. Impact of Gastric Microenvironment Modulation on Semaglutide Delivery

While C10 has traditionally been regarded as having a weaker alkalizing potential compared to SNAC, the results of this study challenge the assumption that this difference limits its clinical utility. Given that the buffering capacity of the fasted human and canine stomach is relatively low, we hypothesized that C10 could provide sufficient neutralization to create a favorable absorption window. The preformulation data (Table 4) validated this hypothesis, demonstrating that C10 at 300 mg can effectively elevate the local pH to 6.22, a critical threshold for semaglutide delivery.

This pH modulation serves a dual purpose that is essential for overcoming the formidable barriers to oral peptide absorption. First, it addresses the solubility hurdle of semaglutide, which is notoriously poorly soluble in the acidic range of pH 2.0 to 5.0. By shifting the environment above pH 6.0, C10 ensures that the peptide is fully solubilized and available for uptake. Simultaneously, this alkaline shift is vital for the enhancer’s own functionality. With a *pK_a_* of approximately 4.8, C10 must remain in its ionized (salt) form to act as a permeation modulator. At the established pH of 6.22, C10 is predominantly ionized, preventing its precipitation into insoluble capric acid and maintaining a high effective concentration at the site of action.

Furthermore, the present study highlights that the physical state of the C10 dispersion is as decisive as its chemical ionization. Effective permeation enhancement by MCFAs is known to be driven by free monomers and small, dynamic micellar structures that can readily partition into the epithelial membrane [17,36,37]. Our DLS analysis (Table 5) revealed that the 300 mg dose resides in an optimal physicochemical range, maintaining a fine sub-micron dispersion (approximately 273 nm). Interestingly, while increasing the dose to 500 mg provided a higher pH, it triggered massive aggregation (approximately 17,642 nm). This excessive aggregation, likely due to high ionic strength, sequesters the active species into inactive clusters and depletes the concentration of free monomers available for membrane interaction [37].

These integrated insights explain why 300 mg was identified as the optimal dose for our SSL-T formulation. By simultaneously satisfying the chemical requirement for ionization and the physical requirement for fine dispersion, this rationally designed microenvironment enabled the C10-based system to achieve systemic exposure in beagle dogs comparable to the clinically established SNAC-based reference product.

### 4.3. In Vitro–In Vivo Translation of C10-Mediated Absorption Enhancement

The in vivo pharmacokinetic results further support the functional relevance of C10 as a permeation enhancer under physiological conditions. In Sprague–Dawley rats, C10 increased systemic exposure to semaglutide in a clear dose-dependent manner, indicating that MCFA-mediated enhancement is maintained after oral administration. The pronounced increase in exposure at higher C10 loadings suggests a threshold-like effect, likely reflecting the need to achieve a sufficient local enhancer concentration at the epithelial interface. This interpretation is consistent with the non-linear enhancer–membrane interactions reported for fatty acid-based permeation enhancers. At equivalent enhancer doses, C10 produced systemic exposure comparable to that achieved with SNAC. The overall agreement between the in vitro permeability trends and the in vivo rat pharmacokinetic data supports the view that the observed enhancement is mechanistically meaningful rather than a model-specific artifact [38]. Collectively, these findings suggest that the in vitro observations capture key features relevant to in vivo absorption behavior.

### 4.4. Influence of Formulation Architecture on Dissolution and Absorption

The three IR tablet architectures evaluated in this study—single-layer (SSL-T), bilayer (SBL-T), and compression-coated (SCC-T)—enabled a systematic assessment of how formulation structure influences dissolution behavior and enhancer deployment. All formulations achieved rapid drug release across pH 1.2, 4.0, and 6.8, consistent with the requirements for IR oral semaglutide delivery. SSL-T and SBL-T exhibited dissolution profiles comparable to those of the reference drug product (Rybelsus), as supported by f_2_ similarity factors greater than 50. These results suggest reference-like early-stage release and rapid availability of semaglutide in the dissolution medium. In contrast, SCC-T showed a short initial lag phase, most likely due to hydration and disintegration of the outer compression-coated layer before exposure of the drug-containing core.

Although SCC-T eventually achieved a comparable extent of dissolution, this early delay affected the early-stage release profile and resulted in a non-similar f_2_ value compared with the reference product. These findings indicate that, for the present oral semaglutide formulation, rapid co-release of semaglutide and C10 is more favorable than delayed or staged release, because prompt local availability of both the peptide and enhancer is required to establish the intended absorption-enhancing microenvironment.

From a formulation perspective, the overall rapid dissolution observed across the different architectures highlights the flexibility of C10-based systems and their adaptability to various manufacturing strategies, although early-stage release characteristics can be influenced by the spatial arrangement of the drug-containing and enhancer-containing layers [39,40].

### 4.5. Cross-Species Pharmacokinetic Performance and Clinical Implications

The beagle dog pharmacokinetic results further support the translational potential of the optimized C10-based tablet formulation. In this larger animal model, the plasma concentration–time profile of semaglutide following administration of the optimized formulation was broadly comparable to that of the SNAC-based reference product, with similar *C*_max_, *AUC*_last_, and elimination half-life values. These findings indicate that the C10-based IR system was able to reproduce systemic exposure at a level comparable to the reference product under physiologically more demanding conditions.

This observation is particularly relevant because oral absorption of peptide therapeutics is generally more challenging and variable in larger species than in rodents. In this context, the beagle dog data provide important support for the translational robustness of the formulation strategy identified through the preceding preformulation, in vitro, and rat in vivo studies. The comparable exposure achieved in dogs suggests that the C10-based system can maintain its functional performance beyond proof-of-concept conditions and within a formulation format more relevant to eventual clinical application. At the same time, the present findings should be interpreted within the known variability of oral peptide delivery systems employing permeation enhancers. Considerable inter-animal variability was observed in both treatment groups, which is consistent with the pharmacokinetic behavior commonly reported for this class of formulations.

Nevertheless, the overall overlap in exposure and disposition characteristics between the optimized C10-based tablet and the reference product supports the feasibility of C10 as an alternative enhancer platform for oral semaglutide delivery.

### 4.6. Study Limitations and Future Directions

Several limitations of the present study should be acknowledged. First, the Caco-2 model used for permeability assessment does not fully capture the complexity of the gastrointestinal environment in vivo, including mucus, luminal dilution, regional differences in absorption, and dynamic fluid movement. Second, although the rat and beagle dog studies provided useful pharmacokinetic information, species-specific differences in gastrointestinal physiology may limit direct extrapolation of these findings to humans. Third, while no abnormal clinical signs were observed following single-dose administration in the animal studies, the long-term safety and tolerability of repeated exposure to high levels of C10 were not evaluated. Another important consideration for MCFA-based permeation enhancement is the potential role of bile acids and bile salts in the intestinal lumen. Under physiological conditions, fatty acid-based enhancers such as C10 may interact with endogenous bile salts and form mixed colloidal or micellar structures, which can influence the apparent solubility, free monomer concentration, membrane partitioning, and consequently the permeation-enhancing activity of the enhancer. Therefore, the enhancer performance observed under simplified in vitro or gastric-relevant conditions may not fully represent the dynamic intestinal environment where bile salts are present. Although the present study focused primarily on the gastric microenvironment and the systemic performance of C10-based immediate-release tablets, the interaction between C10, bile salts, and semaglutide should be further investigated using biorelevant intestinal media or bile salt-containing in vitro models. Such studies would help clarify how endogenous bile components influence the availability and absorption-enhancing function of C10 in vivo. Future studies should therefore focus on strengthening both the mechanistic and translational understanding of this formulation system. Additional approaches, such as in situ perfusion studies and physiologically based pharmacokinetic modeling, may help to better define the relationship between local gastrointestinal conditions and semaglutide absorption. In parallel, dedicated repeat-dose safety studies, including histopathological evaluation, will be needed to further characterize the tolerability of C10-containing formulations under conditions more relevant to chronic clinical use. Despite these limitations, the present findings provide a coherent basis for continued development of C10-based oral semaglutide formulations. The combined preformulation, in vitro, and in vivo results support the view that C10 can serve as a viable alternative enhancer platform when incorporated into an appropriately designed IR system.

## 5. Conclusions

This study demonstrates that C10, an MCFA-derived permeation enhancer, is a mechanistically and pharmaceutically relevant approach for improving the oral absorption of semaglutide. The preformulation results showed that C10 can establish a favorable gastric microenvironment for semaglutide delivery by modulating local pH while maintaining a physicochemically suitable dispersed state, supporting its dual role in both drug solubilization and absorption enhancement. In vitro permeability studies and rat pharmacokinetic data further showed that C10-mediated enhancement was concentration-dependent and remained functionally relevant after oral administration. Among the tablet architectures evaluated, the IR single-layer design provided the most appropriate balance of reference-like dissolution behavior, mechanistic simplicity, and practical suitability for further development. The optimized C10-based tablet achieved systemic exposure comparable to that of the SNAC-based reference product in beagle dogs under the tested conditions, supporting the translational potential of this formulation strategy. Taken together, these findings support the feasibility of C10 as an alternative enhancer platform for oral semaglutide delivery and provide a basis for further formulation optimization and preclinical development of C10-based oral peptide systems. Building on these results, our ongoing research is further investigating how different alkalizing agents influence the local gastric microenvironment and oral absorption of semaglutide, with the aim of expanding the applicability of MCFA-based enhancer systems for oral peptide delivery.

## Figures and Tables

**Table 1:** Composition of semaglutide tablets.

Layer Components	SSL-T	Amount (mg/Tablet)
Single-layer	Semaglutide	14.0
	Lactose monohydrate	300.0
	Copovidone	13.0
	Sodium caprate	300.0
	Croscarmellose sodium	17.0
	Magnesium stearate	8.0
	Total weight	652.0

Abbreviations: SSL-T, semaglutide single-layer tablet.

**Table 2:** Composition of semaglutide bilayer tablets.

Layer Components	SBL-T	Amount (mg/Tablet)
Upper layer	Semaglutide	14.0
	Lactose monohydrate	70.0
	Microcrystalline Cellulose	100.0
	Povidone K90	5.0
	Magnesium stearate	2.0
	Total	191.0
Lower layer	Sodium caprate	300.0
	Lactose monohydrate	200.0
	Crospovidone	30.4
	Copovidone VA64	12.6
	Magnesium stearate	5.5
	Total	578.5

Abbreviations: SBL-T, semaglutide bilayer tablet.

**Table 3:** Composition of semaglutide dry compression-coated tablets.

Layer Components	SCC-T	Amount (mg/Tablet)
Core tablet	Semaglutide	14.0
	Microcrystalline Cellulose	77.0
	Crospovidone	5.0
	Povidone K90	2.0
	Magnesium stearate	2.0
	Total	100.0
Outer layer	C10	300.0
	Lactose monohydrate	330.0
	Crospovidone	30.4
	Copovidone VA64	12.6
	Magnesium stearate	7.0
	Total	680.0

Abbreviations: SCC-T, semaglutide dry compression-coated tablet.

**Table 4:** Acid-neutralizing capacity of C10 under simulated gastric conditions for beagle dogs and humans.

Parameter	Simulated Beagle Gastric Condition			Simulated Human Gastric Condition		
	C10 100 mg	C10 300 mg	C10 500 mg	C10 100 mg	C10 300 mg	C10 500 mg
pH (15 min)	6.23	7.16	7.48	4.92	6.16	6.46
pH (45 min)	6.32	7.23	7.53	4.98	6.22	6.51

**Table 5:** Particle size characteristics and distribution of C10 dispersions under gastric-relevant conditions.

Parameter	Simulated Beagle Gastric Condition			Simulated Human Gastric Condition		
	C10 100 mg	C10 300 mg	C10 500 mg	C10 100 mg	C10 300 mg	C10 500 mg
*Z*-average (nm)	2838.3	161.6	206.2	125.7	273.9	17,642.2
PDI	1.5	0.29	0.30	0.15	0.19	3.97
*D*_10_ (nm)	159.7	73.0	80.0	73.2	113.7	25,443.8
*D*_50_ (nm)	57,183.5	172.9	252.2	106.7	150.6	91,171.1
*D*_90_ (nm)	92,766.4	515.1	634.9	165.5	3587.5	126,250.3

Abbreviations: *Z*-average, intensity-weighted harmonic mean size; PDI, polydispersity index; *D*_10_, *D*_50_, and *D*_90_, particle diameters at 10%, 50%, and 90% of the cumulative distribution, respectively.

**Table 6:** Permeation-enhancing effects of C10 and SNAC on semaglutide across Caco-2 monolayers.

Solvent	*P_app_* (cm/s)	Fold Change
Control (semaglutide alone)	2.49 × 10^−7^	-
C10 (2.5 mM)	3.30 × 10^−7^	1.32
C10 (5 mM)	3.58 × 10^−7^	1.43
C10 (10 mM)	1.57 × 10^−6^	6.30
SNAC (10 mM)	6.67 × 10^−7^	2.67
SNAC (20 mM)	8.19 × 10^−7^	3.28

Abbreviations: *P_app_*, apparent permeability coefficient.

**Table 7:** Pharmacokinetic parameters of semaglutide following oral administration in Sprague–Dawley rats (*n* = 5).

PK Parameter	PK Parameter (Mean ± *SD*)					
	Group 1 DW	Group 2 Semaglutide	Group 3 Semaglutide + C10 100 mg/kg	Group 4 Semaglutide + C10 200 mg/kg	Group 5 Semaglutide + C10 300 mg/kg	Group 6 Semaglutide + SNAC 200 mg/kg
*t*_max_ (h)	N/A	3.00 ± 2.65	1.25 ± 0.87	1.80 ± 1.44	1.70 ± 1.40	1.50 ± 1.54
*C*_max_ (ng/mL)	N/A	9.92 ± 13.42	23.30 ± 39.08	30.39 ± 53.15	171.15 ± 201.29	43.45 ± 44.41
*AUC*_last_ (ng·h/mL)	N/A	58.88 ± 76.38	230.46 ± 431.57	346.90 ± 719.77	1966.51 ± 2507.23	503.09 ± 586.33
*AUC*_inf_ (ng·h/mL)	N/A	211.58	482.01 ± 652.84	630.45 ± 981.87	2266.95 ± 2882.10	939.60 ± 618.33
*t*_1/2_ (h)	N/A	4.38	4.70 ± 2.31	4.07 ± 2.17	5.86 ± 2.13	7.23 ± 0.74

Abbreviations: *SD*, standard deviation; DW, distilled water; PK, pharmacokinetic; SNAC, sodium *N*-[8-(2-hydroxybenzoyl)amino]caprylate; *t*_max_, time to maximum plasma concentration; *C*_max_, maximum plasma concentration; *AUC*_last_, area under the plasma concentration–time curve to last measurable concentration; *AUC*_inf_, area under the concentration–time curve extrapolated to infinity; *t*_1/2_, elimination half-life.

**Table 8:** Pharmacokinetic parameters of semaglutide following oral administration of the reference drug product (Rybelsus) and test tablet (SSL-T) in beagle dogs.

PK Parameter	PK Parameter (Mean ± *SD*)		
	Reference Group (*n* = 5)	Test Group (*n* = 4)	Relative BE (%)
*t*_max_ (h)	3.00 ± 1.00	2.00 ± 0.82	-
*C*_max_ (ng/mL)	26.13 ± 37.56	23.84 ± 25.65	91.2
*AUC*_last_ (ng·h/mL)	712.03 ± 995.25	619.96 ± 697.68	87.1
*AUC*_inf_ (ng·h/mL)	1340.95 ± 1845.80	1187.67 ± 1464.82	88.6
*t*_1/2_ (h)	42.30 ± 24.82	42.32 ± 8.28	-

Abbreviations: *SD*, standard deviation; PK, pharmacokinetic; BE, bioequivalence; *t*_max_, time to maximum plasma concentration; *C*_max_, maximum plasma concentration; *AUC*_last_, area under the plasma concentration–time curve to last measurable concentration; *AUC*_inf_, area under the concentration–time curve extrapolated to infinity; *t*_1/2_, elimination half-life. *Note:* Relative bioequivalence (BE) was calculated based on *AUC* and *C*_max_.

## Data Availability

The original contributions presented in this study are included in the article. Further inquiries can be directed to the corresponding author.

## Supplementary Materials

The following supporting information can be downloaded at: https://www.mdpi.com/article/10.3390/pharmaceutics18060680/s1. Figure S1: Concentration-dependent cytotoxicity of C10 and SNAC measured using MTT and LDH assays. Abbreviations: MTT, 3-(4,5-dimethylthiazol-2-yl)-2,5-diphenyltetrazolium bromide; LDH, lactate dehydrogenase, TX-100, Triton X-100.

## Abbreviations

AUC_inf_	Area under the plasma concentration–time curve extrapolated to infinity
AUC_last_	Area under the plasma concentration–time curve to the last measurable concentration
BE	Bioequivalence
BQL	Below the quantification limit
Caco-2	Human colorectal adenocarcinoma cell line (Caco-2)
C10	Sodium caprate
*C*_max_	Maximum plasma concentration
DLS	Dynamic light scattering
DW	Distilled water
*D*_10_	Particle diameter at 10% of the cumulative distribution
*D*_50_	Particle diameter at 50% of the cumulative distribution
*D*_90_	Particle diameter at 90% of the cumulative distribution
EMA	European Medicines Agency
FDA	Food and Drug Administration
*f*_2_	Similarity factor
GI	Gastrointestinal
GLP-1 RA	Glucagon-like peptide-1 receptor agonist
HCl	Hydrochloric acid
HPLC	High-performance liquid chromatography
IACUC	Institutional Animal Care and Use Committee
IR	Immediate-release
K_2_-EDTA	Dipotassium ethylenediaminetetraacetic acid
*k*_el_	Terminal elimination rate constant
LC–MS/MS	Liquid chromatography–tandem mass spectrometry
LDH	Lactate dehydrogenase
LLOQ	Lower limit of quantification
LSD	Least significant difference
MCC	Microcrystalline cellulose
MCFA	Medium-chain fatty acid
MFDS	Ministry of Food and Drug Safety
MRM	Multiple-reaction monitoring
MTT	3-(4,5-Dimethylthiazol-2-yl)-2,5-diphenyltetrazolium bromide
NCA	Non-compartmental analysis
OM	Optical microscopy
*P_app_*	Apparent permeability coefficient
PDI	Polydispersity index
PK	Pharmacokinetic(s)
QbD	Quality by design
SBL-T	Semaglutide bilayer tablet
SCC-T	Semaglutide compression-coated tablet
*SD*	Standard deviation
SNAC	Sodium *N*-[8-(2-hydroxybenzoyl)amino]caprylate
SSL-T	Semaglutide single-layer tablet
*t*_½_	Elimination half-life
*t*_max_	Time to maximum plasma concentration
TEER	Transepithelial electrical resistance
USP	United States Pharmacopeia

## References

[1] Ahmann A.J., Capehorn M., Charpentier G., Dotta F., Henkel E., Lingvay I., Holst A.G., Annett M.P., Aroda V.R. (2017). Efficacy and Safety of Once-Weekly Semaglutide Versus Exenatide ER in Subjects With Type 2 Diabetes (SUSTAIN 3): A 56-Week, Open-Label, Randomized Clinical Trial. Diabetes Care.

[2] Buckley S.T., Bækdal T.A., Vegge A., Maarbjerg S.J., Pyke C., Ahnfelt-Rønne J., Madsen K.G., Schéele S.G., Alanentalo T., Kirk R.K. (2018). Transcellular stomach absorption of a derivatized glucagon-like peptide-1 receptor agonist. Sci. Transl. Med..

[3] Granhall C., Søndergaard F.L., Thomsen M., Anderson T.W. (2018). Pharmacokinetics, Safety and Tolerability of Oral Semaglutide in Subjects with Renal Impairment. Clin. Pharmacokinet..

[4] Wilding J.P.H., Batterham R.L., Calanna S., Davies M., Gaal L.F.V., Lingvay I., McGowan B.M., Rosenstock J., Tran M.T.D., Wadden T.A. (2021). Once-Weekly Semaglutide in Adults with Overweight or Obesity. N. Engl. J. Med..

[5] Morishita M., Peppas N.A. (2006). Is the oral route possible for peptide and protein drug delivery?. Drug Discov. Today.

[6] Hamman J.H. (2010). Chitosan based polyelectrolyte complexes as potential carrier materials in drug delivery systems. Mar. Drugs.

[7] Lau J., Bloch P., Schäffer L., Pettersson I., Spetzler J., Kofoed J., Madsen K., Knudsen L.B., McGuire J., Steensgaard D.B. (2015). Discovery of the Once-Weekly Glucagon-Like Peptide-1 (GLP-1) Analogue Semaglutide. J. Med. Chem..

[8] Zhu Q., Chen Z., Paul P.K., Lu Y., Wu W., Qi J. (2021). Oral delivery of proteins and peptides: Challenges, status quo and future perspectives. Acta Pharm. Sin. B.

[9] Kim D.-H., Kim J.-E. (2025). Recent advances and trends in oral absorption enhancements of GLP-1 receptor agonist formulations. J. Pharm. Investig..

[10] Twarog C., Fattah S., Heade J., Maher S., Fattal E., Brayden D.J. (2019). Intestinal Permeation Enhancers for Oral Delivery of Macromolecules: A Comparison between Salcaprozate Sodium (SNAC) and Sodium Caprate (C_10_). Pharmaceutics.

[11] Solis-Herrera C., Kane M.P., Triplitt C. (2024). Current Understanding of Sodium N-(8-[2-Hydroxylbenzoyl] Amino) Caprylate (SNAC) as an Absorption Enhancer: The Oral Semaglutide Experience. Clin. Diabetes.

[12] Maher S., Mrsny R.J., Brayden D.J. (2016). Intestinal permeation enhancers for oral peptide delivery. Adv. Drug Deliv. Rev..

[13] McCartney F., Gleeson J.P., Brayden D.J. (2016). Safety concerns over the use of intestinal permeation enhancers: A mini-review. Tissue Barriers.

[14] Maher S., Leonard T., Jacobsen J., Brayden D. (2009). Safety and efficacy of sodium caprate in promoting oral drug absorption: From in vitro to the clinic. Adv. Drug Deliv. Rev..

[15] Kim J.C., Park E.J., Na D.H. (2022). Gastrointestinal Permeation Enhancers for the Development of Oral Peptide Pharmaceuticals. Pharmaceuticals.

[16] Niu Z., La Zara D., Blaabjerg L., Pessi J., Raptis K., Toftlev A., Sauter M., Christophersen P., Bardonnet P.-L., Andersson V. (2025). Combining SNAC and C10 in oral tablet formulations for gastric peptide delivery: A preclinical and clinical study. J. Control. Release.

[17] Lindmark T., Nikkilä T., Artursson P. (1995). Mechanisms of absorption enhancement by medium chain fatty acids in intestinal epithelial Caco-2 cell monolayers. J. Pharmacol. Exp. Ther..

[18] Baral K.C., Choi K.Y. (2025). Barriers and Strategies for Oral Peptide and Protein Therapeutics Delivery: Update on Clinical Advances. Pharmaceutics.

[19] Hens B., Tsume Y., Bermejo M., Paixao P., Koenigsknecht M.J., Baker J.R., Hasler W.L., Lionberger R., Fan J., Dickens J. (2017). Low Buffer Capacity and Alternating Motility along the Human Gastrointestinal Tract: Implications for in Vivo Dissolution and Absorption of Ionizable Drugs. Mol. Pharm..

[20] Mudie D.M., Amidon G.L., Amidon G.E. (2010). Physiological parameters for oral delivery and in vitro testing. Mol. Pharm..

[21] Lui C.Y., Amidon G.L., Berardi R.R., Fleisher D., Youngberg C., Dressman J.B. (1986). Comparison of gastrointestinal pH in dogs and humans: Implications on the use of the beagle dog as a model for oral absorption in humans. J. Pharm. Sci..

[22] Bhattachar S.N., Perkins E.J., Tan J.S., Burns L.J. (2011). Effect of gastric pH on the pharmacokinetics of a bcs class II compound in dogs: Utilization of an artificial stomach and duodenum dissolution model and gastroplus,^TM^ simulations to predict absorption. J. Pharm. Sci..

[23] Kang S.-J., Kim J.-E. (2023). Development of Clinically Optimized Sitagliptin and Dapagliflozin Complex Tablets: Pre-Formulation, Formulation, and Human Bioequivalence Studies. Pharmaceutics.

[24] Yoon J.-H., Kim D.-H., Kim J.-E. (2026). Quality by Design-Based Formulation Development of an Oral Semaglutide Tablet. Pharmaceutics.

[25] Wang C., Zhai B., Guo H., Wang P., Liu Z., Gu H., Ho H., Langguth P., Li K., Wang C. (2020). In vivo measurement of gastric fluid volume in anesthetized dogs. J. Drug Deliv. Sci. Technol..

[26] Roelofs J.J.M., Camps G., Leenders L.M., Marciani L., Spiller R.C., Van Eijnatten E.J.M., Alyami J., Deng R., Freitas D., Grimm M. (2024). Intra- and interindividual variability in fasted gastric content volume. Neurogastroenterol. Motil..

[27] Mudie D.M., Murray K., Hoad C.L., Pritchard S.E., Garnett M.C., Amidon G.L., Gowland P.A., Spiller R.C., Amidon G.E., Marciani L. (2014). Quantification of Gastrointestinal Liquid Volumes and Distribution Following a 240 mL Dose of Water in the Fasted State. Mol. Pharm..

[28] Khalil A.M., Ragab S.G., Botros J.M., Abd-Aal H.A., Boules M.L. (2021). Gastric residual volume assessment by gastric ultrasound in fasting obese patients: A comparative study. Anesthesiol. Pain Med..

[29] U.S. Food and Drug Administration RYBELSUS^®^ (Semaglutide) Tablets, for Oral Use: Prescribing Information. https://www.accessdata.fda.gov/drugsatfda_docs/label/2024/213051s020s021lbl.pdf.

[30] Lee Y.-J., Kim J.-E. (2022). In Vitro–In Vivo Correlation of Tianeptine Sodium Sustained-Release Dual-Layer Tablets. Molecules.

[31] Kang S.-J., Kim J.-E. (2023). The Development and Validation of Simultaneous Quantitative Analysis Reversed-phase High-performance Liquid Chromatography for Sitagliptin Phosphate Monohydrate and Dapagliflozin Propanediol Monohydrate Fixed-dose Combination Dual-layered Tablet. Curr. Pharm. Anal..

[32] Lee S.-H., Kim J.-E. (2021). Quality by Design Applied Development of Immediate-Release Rabeprazole Sodium Dry-Coated Tablet. Pharmaceutics.

[33] Twarog C., Liu K., O’Brien P.J., Dawson K.A., Fattal E., Illel B., Brayden D.J. (2020). A head-to-head Caco-2 assay comparison of the mechanisms of action of the intestinal permeation enhancers: SNAC and sodium caprate (C10). Eur. J. Pharm. Biopharm..

[34] Hubatsch I., Ragnarsson E.G., Artursson P. (2007). Determination of drug permeability and prediction of drug absorption in Caco-2 monolayers. Nat. Protoc..

[35] Weerink M.A.S., Struys M., Hannivoort L.N., Barends C.R.M., Absalom A.R., Colin P. (2017). Clinical Pharmacokinetics and Pharmacodynamics of Dexmedetomidine. Clin. Pharmacokinet..

[36] Anderberg E.K., Lindmark T., Artursson P. (1993). Sodium Caprate Elicits Dilatations in Human Intestinal Tight Junctions and Enhances Drug Absorption by the Paracellular Route. Pharm. Res..

[37] Berg S., Kärrberg L., Suljovic D., Seeliger F., Söderberg M., Perez-Alcazar M., Van Zuydam N., Abrahamsson B., Hugerth A.M., Davies N. (2022). Impact of Intestinal Concentration and Colloidal Structure on the Permeation-Enhancing Efficiency of Sodium Caprate in the Rat. Mol. Pharm..

[38] Lee S.-H., Kim J.-K., Jee J.-P., Jang D.-J., Park Y.-J., Kim J.-E. (2022). Quality by Design (QbD) application for the pharmaceutical development process. J. Pharm. Investig..

[39] Gong S.-J., Kim H.R., Park Y.-J., Kim J.-E. (2025). Recent trends in continuous manufacturing and digitalization in pharmaceutical process development and manufacturing. J. Pharm. Investig..

[40] Jung E.-A., Park Y.-J., Kim J.-E. (2023). Application of continuous manufacturing for solid oral dosage forms. J. Pharm. Investig..

