# Supplementary material for: Formulation Engineering of Oral Semaglutide Tablets: Unleashing Gastric Intestinal Permeation with Sodium Caprate
Source: Pharmaceutics. 2026 May 29;18(6):680. doi: 10.3390/pharmaceutics18060680 (PMC13306039; doi:10.3390/pharmaceutics18060680)
Supplement: Supplementary file 1 [file pharmaceutics-18-00680-s001.zip › pharmaceutics-4319690-supplementary.pdf]

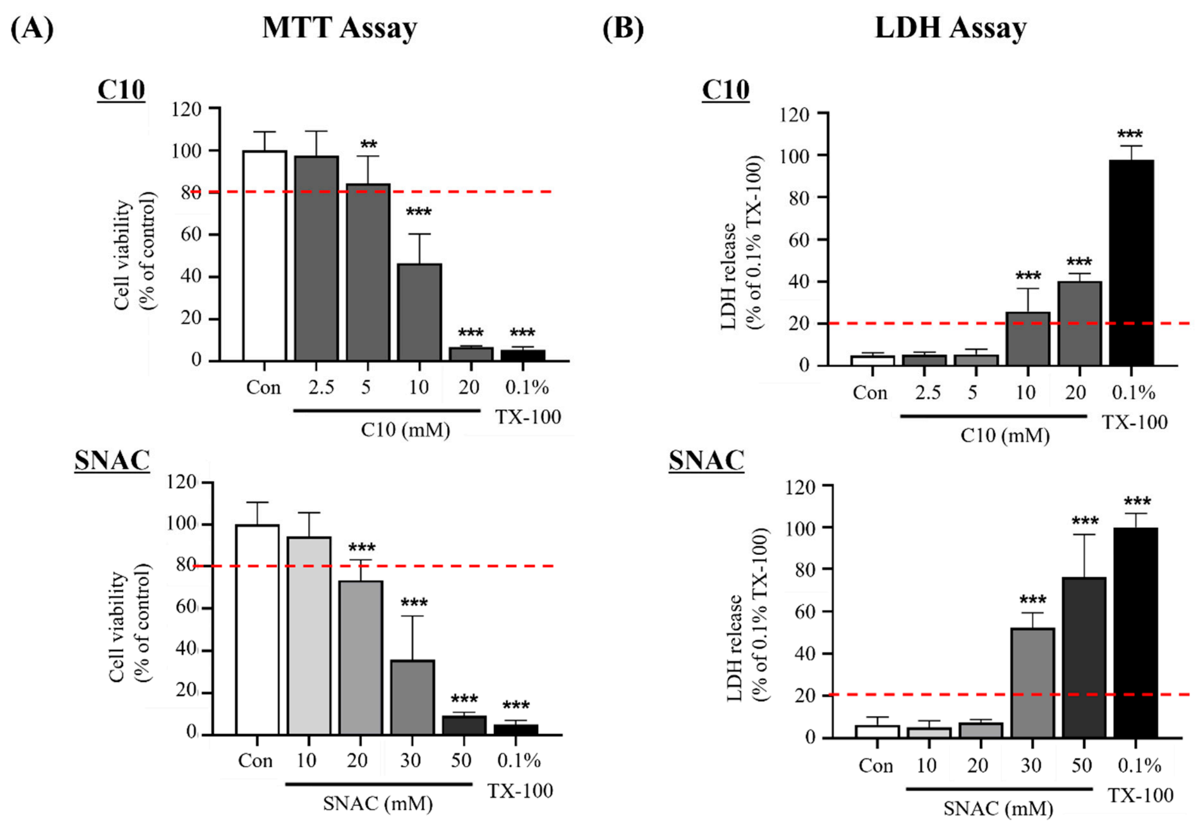


**Figure S1.** Concentration-dependent cytotoxicity of C10 and SNAC measured using MTT and LDH assays.

Abbreviations: MTT, 3-(4,5-dimethylthiazol-2-yl)-2,5-diphenyltetrazolium bromide; LDH, lactate dehydrogenase, TX-100, Triton X-100
